# Supplementary material for: Phylogeographic and Paleoclimatic Modelling Tools Improve Our Understanding of the Biogeographic History of Hierophis viridiflavus (Colubridae)
Source: Animals (Basel). 2023 Jun 29;13(13):2143. doi: 10.3390/ani13132143 (PMC10340045; doi:10.3390/ani13132143)
Supplement: Supplementary file 1 [file animals-13-02143-s001.zip › animals-2449156 - supplementary material (updated).pdf]

Supplementary material

Supplementary text

Supplementary Text S1. Details on DNA extraction and sequencing for 80 unpublished sequences generated in this work

Total genomic DNA was extracted from ethanol-preserved tissues using BioSprint 15 DNA Blood Kit following the manufacturer's protocol (Qiagen, Hilden, Germany). The NADH dehydrogenase subunit 4 (ND4) was amplified for all samples by standard polymerase chain reaction (PCR), using the primer ND4-Leu [1]. In some cases, DNA amplification was not possible with this primer and thus, we used the primers ND4HvF-ND4HvR [2].

PCRs were performed in a total volume of 25µl and included 17.8µl of H_2O, 2.5µl of a reaction buffer x 10, 1µl of dNTP (10mM), 1.5µl of $MgCl_2$ (50mM), 0.5µl of each primer (10mM), 0.2µl of DNA polymerase and 1µl of template DNA. The thermocycling conditions consisted of an initial denaturation at 94°C for 5 min, 40 cycles at 94°C for 30 s, 50°C for 40 s and 72°C for 45 s followed by a final step at 72°C for 10 min. PCR products were checked on 1% agarose gels, and the products of expected length were directly sequenced at Macrogen Inc (Macrogen Europe).

References

1. Arévalo, E.; Davis, S.K.; Sites, J.W.Jr. Mitochondrial DNA sequence divergence and phylogenetic relationships among eight chromosome races of the *Sceloporus grammicus* complex (Phrynosomatidae) in Central Mexico. *Syst. Biol*. **1994**, *43*, 387–418.
2. Mezzasalma, M.; Dall'Asta, A.; Loy, A.; Cheylan, M.; Lymberakis, P.; Zuffi, M.A.L.; Tomovic, L.; Odierna, G.; Guarino, F.M. A sisters' story: Comparative phylogeography and taxonomy of *Hierophis viridiflavus* and *H. gemonensis* (Serpentes, Colubridae). *Zool. Scr*. **2015**, *44*, 495–508.

Supplementary tables

Table S1. List of the analyzed samples with codes, linage/sublinage assignment, localities, coordinates (latitude and longitude), corresponding haplotype, GenBank accession number, publication, collection and number. Samples in bold correspond to the ones which are specific of this study. TBA means to be added after acceptance.

**code**	**Linage/Sublinage**	**Locality**	**latitude**	**longitude**	***Haplotype***	***Accession numbers***	***Publication***	***Collection***	***Coll. number***
RV01	*East/South-east-north*	San Vito (Treviso, Italy)	45.898	11.953	H32	MW297621	1		/
RV02	*East/South-east-north*	Possagno (Treviso, Italy)	45.850	11.874	H32	MW297622	1		/
RTR01	*East/South-east-north*	Pergine Valsugana (Trento, Italy)	46.059	11.241	H32	MW297618	1		/
RPT03	*West*	Noasca (Torino, Italy)	45.454	7.317	H1	MW297556	1		/
RPT02	*West*	Pianchette (Torino, Italy)	45.452	7.293	H1	MW297555	1		/
RPT01	*West*	Villastellone (Torino, Italy)	44.923	7.742	H1	MW297553	1		/
RPT04	*West*	Fornolosa (Torino, Italy)	45.431	7.399	H1	MW297557	1		/
RLO01	*West*	Pavia (Italy)	45.209	9.167	H1	MW297558	1		/
RLO02	*East/South-east-north*	Le Bine. Calvatone (Cremona, Italy)	45.132	10.442	H32	MW297619	1		/
RLO03	*East/South-east-north*	Le Bine. Calvatone (Cremona, Italy)	45.132	10.442	H32	MW297620	1		R3 (2)

RE11	*East/South-east-north*	La Foscarina (Rovigo, Italy)	44.944	11.620	H32	MW297623	1	/
RE12	*West*	Casalecchio (Rimini, Italy)	43.836	12.268	H1	MW297561	1	R3 (2)
RE13	*West*	Villa di Sant'Alessio (Forlì-Cesena, Italy)	43.810	11.987	H1	MW297562	1	/
RLI01	*West*	Genova (Italy)	44.394	9.017	H1	MW297559	1	/
RLI02	*West*	Genova (Italy)	44.394	9.017	H1	MW297560	1	/
RT14	*West*	Frosini (Siena, Italy)	43.216	11.155	H1	MW297575	1	R3 (2)
RT15	*West*	Rosia (Siena, Italy)	43.250	11.224	H1	MW297576	1	R3 (2)
RT17	*West*	Baccinello (Grosseto, Italy)	42.796	11.375	H1	MW297578	1	/
RT18	*West*	Campagnatico (Grosseto, Italy)	42.887	11.242	H1	MW297579	1	R2 (2)
RT19	*West*	Petriccio (Grosseto, Italy)	43.079	10.997	H1	MW297580	1	R2 (2)
RT12	*West*	Boccheggiano (Grosseto, Italy)	43.082	11.025	H1	MW297573	1	/
RT13	*West*	Boccheggiano (Grosseto, Italy)	43.082	11.025	H1	MW297574	1	/
RT10	*West*	Prata (Grosseto, Italy)	43.081	10.984	H1	MW297571	1	/
RT11	*West*	Prata (Grosseto, Italy)	43.081	10.984	H1	MW297572	1	/
RT16	*West*	Prata (Grosseto, Italy)	43.081	10.984	H1	MW297577	1	/
RT20	*West*	Prata (Grosseto, Italy)	43.079	10.988	H1	MW297581	1	/
RT21	*West*	Tatti (Grosseto, Italy)	43.025	11.029	H1	MW297582	1	R2 (2)
RT22	*West*	Camaldoli (Arezzo, Italy)	43.794	11.820	H1	MW297583	1	R3 (2)

RT23	*West*	Sgrillozzo (Grosseto, Italy)	42.537	11.406	H1	MW297584	1	R2 (2)
RT24	*West*	Massa Marittima (Grosseto, Italy)	43.051	10.889	H1	MW297585	1	/
RT25	*West*	Portiglione (Grosseto, Italy)	42.881	10.781	H1	MW297586	1	R2 (1). R4 (1)
RT26	*West*	Follonica (Grosseto, Italy)	42.930	10.762	H1	MW297587	1	R2 (2)
RT27	*West*	Pian D'Alma (Grosseto, Italy)	42.856	10.775	H1	MW297588	1	R2 (1). R4 (1)
RT29	*West*	Cerboli (Livorno, Italy)	42.858	10.547	H1	MW297589	1	R2 (2)
RT30	*West*	Firenze (Italy)	43.785	11.218	H1	MW297590	1	R2 (1). R4 (1)
RT31	*West*	Siena (Italy)	43.314	11.330	H2	MW297591	1	R3 (2)
RT53	*West*	Podere Corso (Grosseto, Italy)	42.659	11.238	H1	MW297592	1	/
RMA01	*West*	Torrette (Pesaro e Urbino, Italy)	43.793	13.085	H1	MW297563	1	R2 (2)
RMA02	*West*	Abbadia di Fiastra (Macerata, Italy)	43.228	13.430	H1	MW297564	1	R2 (2)
RSA08	*West*	Limbara (Olbia-Tempio, Italy)	40.856	9.158	H1	MW297568	1	/
RSA03	*West*	Alghero (Italy)	40.587	8.370	H1	MW297565	1	R3 (2)
RSA07	*West*	Alghero (Italy)	40.587	8.370	H1	MW297566	1	R3 (2)
RSA09	*West*	Alghero (Italy)	40.587	8.370	H1	MW297567	1	/
RSA12	*West*	Monti (Olbia-Tempio, Italy)	40.805	9.321	H11	MW297569	1	/

RSA21	*West*	Aglientu (Olbia-Tempio, Italy)	41.079	9.114	H1	MW297570	1	/
RU03	*West*	Città di Castello (Perugia, Italy)	43.496	12.249	H1	MW297593	1	R2 (2)
RU04	*West*	Città della Pieve (Perugia, Italy)	42.947	12.006	H3	MW297594	1	R3 (2)
RL77	*West*	Rieti (Italy)	42.367	12.898	H1	MW297601	1	/
RL35	*West*	Belmonte Castello (Frosinone, Italy)	41.574	13.800	H1	MW297595	1	R3 (1). R5(1)
RL78	*East/Central*	Puzzali (Frosinone, Italy)	41.311	13.766	H22	MW297616	1	/
RL54	*West*	Itri (Latina, Italy)	41.290	13.532	H1	MW297599	1	R2 (2)
RL36	*West*	Foglino (Roma, Italy)	41.461	12.697	H1	MW297596	1	R2 (1). R4 (1)
RL37	*West*	Foglino (Roma, Italy)	41.461	12.697	H1	MW297597	1	R3 (1). R5(1)
RL76	*West*	Morlupo (Roma, Italy)	42.149	12.503	H1	MW297600	1	R3 (2)
RL45	*West*	Capena (Roma, Italy)	42.141	12.539	H1	MW297598	1	R3 (2)
RL81	*West*	Monti Lepini (Latina, Italy)	41.602	13.094	H1	MW297602	1	R3 (2)
RL209	*West*	Sperlonga (Latina, Italy)	41.247	13.466	H1	MW297603	1	R1 (1). R2 (1)
RL210	*West*	Spadellata (Roma, Italy)	41.514	12.607	H1	MW297604	1	/
RL211	*West*	Spadellata (Roma, Italy)	41.514	12.607	H1	MW297605	1	R3 (2)
RL216	*West*	Monte Piano(Roma, Italy)	41.255	13.471	H1	MW297606	1	/

RL229	*West*	Monte Piano(Roma, Italy)	41.247	13.466	H1	MW297607	1	/
RL230	*West*	Roma (Italy)	41.896	12.577	H1	MW297608	1	R2 (2)
RL234	*West*	Roma (Italy)	41.896	12.577	H1	MW297611	1	R3 (2)
RL231	*West*	Monte Piano(Roma, Italy)	41.247	13.466	H1	MW297609	1	R3 (2)
RL232	*West*	Roma (Italy)	41.935	12.644	H1	MW297610	1	/
RL235	*East/Central*	San Giorgio a Liri (Frosinone, Italy)	41.416	13.764	H21	MW297617	1	R2 (2)
RL236	*West*	Castel Porziano (Roma, Italy)	41.744	12.399	H1	MW297612	1	/
RL237	*West*	Roma (Italy)	41.976	12.483	H5	MW297554	1	R2 (1). R4 (1)
RL238	*West*	Nettuno (Roma, Italy)	41.451	12.764	H1	MW297613	1	/
RL239	*West*	Fregene (Roma, Italy)	41.855	12.198	H1	MW297614	1	R2 (2)
RL240	*West*	Manziana (Roma, Italy)	42.131	12.126	H1	MW297615	1	R2 (2)
RC56	*East/Central*	Ailano (Caserta, Italy)	41.385	14.213	H21	MW297635	1	R1 (1). R2 (1)
RC46	*East/Central*	Piedimonte Matese (Caserta, Italy)	41.388	14.404	H21	MW297625	1	/
RC47	*East/Central*	Monte Orfano (Caserta, Italy)	41.388	14.404	H21	MW297626	1	/
RC50	*East/South-east-north*	Monte Orfano (Caserta, Italy)	41.388	14.404	H32	MW297629	1	R1 (1). R2 (1)
RC48	*East/Central*	San Gregorio Matese (Caserta, Italy)	41.388	14.409	H21	MW297627	1	R1 (1). R4 (1)

RC45	*East/Central*	Piedimonte Matese(Caserta, Italy)	41.348	14.375	H21	MW297624	1	R1 (1). R2 (1)
RC55	*East/Central*	Cusano Mutri (Benevento, Italy)	41.316	14.528	H21	MW297634	1	R3 (2)
RC52	*East/South-east-north*	Postiglione (Salerno, Italy)	40.554	15.216	H32	MW297631	1	/
RC54	*East/South-east-north*	Postiglione (Salerno, Italy)	40.554	15.216	H32	MW297633	1	/
RC51	*East/South-east-north*	Castelcivita (Salerno, Italy)	40.496	15.228	H31	MW297630	1	R1 (1). R2 (1)
RC53	*East/South-east-north*	Monti Alburni (Salerno, Italy)	40.474	15.276	H31	MW297632	1	R4 (2)
RC49	*East/South-east-north*	Monticello (Caserta, Italy)	41.119	14.350	H31	MW297628	1	R3 (2)
RM01	*East/South-east-north*	Longano (Isernia, Italy)	41.523	14.266	H32	MW297636	1	R1 (1). R2 (1)
RM02	*East/South-east-north*	Bojano(Campobasso, Italy)	41.492	14.464	H32	MW297637	1	R1 (1). R2 (1)
RM03	*East/South-east-north*	Colle San Leonardo (Chieti, Italy)	41.568	14.287	H32	MW297638	1	R3 (1). R6 (1)
RB07	*East/South-east-north*	Maratea (Potenza, Italy)	40.071	15.707	H28	MW297649	1	/

RB08	*East/South-east-north*	Lauria (Potenza, Italy)	40.044	15.845	H32	MW297650	1	/
RB09	*East/South-east-north*	Fiumicello Santa Venere (Potenza, Italy)	39.999	15.700	H32	MW297651	1	/
RP09	*East/South-east-north*	Peschici (Foggia, Italy)	41.947	16.013	H32	MW297652	1	R1 (1). R4 (1)
RP17	*East/South-east-north*	Ischitella(Foggia, Italy)	41.906	15.899	H32	MW297653	1	/
RP19	*East/South-east-north*	Torre Dell'Orso (Lecce, Italy)	40.164	18.245	H32	MW297654	1	R1 (2)
RP25	*East/South-east-north*	Uggiano la Chiesa (Lecce, Italy)	40.129	18.473	H30	MW297655	1	/
RP26	*East/South-east-north*	Masseria Cesine. Vernole (Lecce, Italy)	40.347	18.337	H32	MW297656	1	/
RP28	*East/South-east-north*	Ruvo di Puglia(Bari, Italy)	41.034	16.495	H25	MW297657	1	/
RP32	*East/South-east-north*	Masseria Pellicciari(Bari, Italy)	40.875	16.442	H25	MW297660	1	/
RP34	*East/South-east-north*	Castel del Monte (Barletta-Andria-Trani, Italy)	41.072	16.266	H25	MW297661	1	/
RP35	*East/South-east-north*	Zapponeta (Foggia, Italy)	41.435	15.998	H25	MW297662	1	/

RP30	*East/South-east-north*	Invaso del Locone (Barletta-Andria-Trani, Italy)	41.064	16.009	H25	MW297658	1	/
RP36	*East/South-east-north*	Trullo di Sopra (Bari, Italy)	40.972	16.326	H24	MW297663	1	/
RP31	*East/South-east-north*	Mottola (Taranto, Italy)	40.621	17.062	H23	MW297659	1	/
RCL124	*East/South-east-north*	Cetraro (Taranto, Italy)	39.541	15.993	H32	MW297648	1	/
RCL10	*East/Sicily*	Lattarico (Cosenza, Italy)	39.464	16.138	H19	MW297641	1	/
RCL08	*East/South-east-north*	Montalto Uffugo (Cosenza, Italy)	39.407	16.157	H25	MW297640	1	/
RCL123	*East/South-east-north*	Falconara (Cosenza, Italy)	39.275	16.093	H32	MW297647	1	R1 (1). R4 (1)
RCL120	*East/South-east-north*	Fiumefreddo Bruzio (Cosenza, Italy)	39.232	16.073	H32	MW297644	1	/
RCL121	*East/South-east-north*	Fiumefreddo Bruzio (Cosenza, Italy)	39.232	16.073	H32	MW297645	1	/
RCL122	*East/South-east-north*	Fiumefreddo Bruzio (Cosenza, Italy)	39.232	16.073	H32	MW297646	1	/
RCL11	*East/South-east-north*	San Luca (Reggio Calabria, Italy)	38.167	16.000	H32	MW297642	1	/
RCL12	*East/Sicily*	Brancaleone (Reggio Calabria, Italy)	37.971	16.098	H19	MW297643	1	R1 (1). R4 (1)

RCL02	*East/South-east-north*	Palizzi(Reggio Calabria, Italy)	37.966	15.988	H32	MW297639	1	/
RS171	*East/Sicily*	Monti Peloritani (Messina, Italy)	38.050	15.333	H20	MW297674	1	R1 (2)
RS14	*East/Sicily*	Gole Alcantara (Catania, Italy)	37.877	15.177	H14	MW297664	1	R4 (2)
RS43	*East/Sicily*	Saline di Priolo (Siracusa, Italy)	37.146	15.220	H14	MW297667	1	R4 (2)
RS219	*East/Sicily*	Vittoria (Ragusa, Italy)	36.966	14.565	H14	MW297676	1	/
RS162	*East/Sicily*	Manfria (Caltanissetta, Italy)	37.113	14.154	H17	MW297673	1	/
RS27	*East/Sicily*	Scala dei Turchi (Agrigento, Italy)	37.294	13.469	H14	MW297665	1	/
RS28	*East/Sicily*	Scala dei Turchi (Agrigento, Italy)	37.294	13.469	H14	MW297666	1	/
RS156	*East/Sicily*	Siculiana (Agrigento, Italy)	37.339	13.391	H18	MW297672	1	/
RS70	*East/Sicily*	Favignana (Trapani, Italy)	37.945	12.296	H20	MW297671	1	/
RS55	*East/Sicily*	Monte Cofano (Trapani, Italy)	38.099	12.666	H20	MW297670	1	/
RS53	*East/Sicily*	San Vito lo Capo (Trapani, Italy)	38.119	12.734	H20	MW297669	1	R4 (2)
RS253	*East/Sicily*	Cerda (Palermo, Italy)	37.895	13.828	H15	MW297677	1	/
RS45	*East/Sicily*	Cefalù (Palermo, Italy)	38.024	14.055	H14	MW297668	1	R1 (1). R4 (1)

RS269	*East/Sicily*	Monti Nebrodi (Messina, Italy)	37.890	14.652	H14	MW297678	1	/
RS295	*East/Sicily*	Lipari (Messina, Italy)	38.483	14.946	H16	MW297679	1	/
RL79	*West*	Ventotene (Latina, Italy)	40.798	13.432	H1	KY923286	2	/
RL22	*West*	Ponza (Latina, Italy)	40.896	12.959	H1	KY923284	2	/
RL66	*West*	Ponza (Latina, Italy)	40.896	12.959	H1	KY923285	2	/
RL80	*West*	Palmarola (Latina, Italy)	40.936	12.859	H1	KY923287	2	/
RS276	*East/Sicily*	Vulcano (Messina, Italy)	38.420	14.958	H14	KY923282	2	/
RS85	*East/Sicily*	Favignana (Trapani, Italy)	37.945	12.296	H20	KY923281	2	/
RS296	*East/Sicily*	Ragusa (Italy)	36.926	14.725	H14	KY923283	2	/
Hv15	*East/South-east-north*	Padriciano (Italy)	45.641	13.827	H25	LN552059	3	/
Hv33	*East/South-east-north*	Talponedo (Italy)	45.722	12.469	H32	LN552077	3	/
Hv17	*East/South-east-north*	Pavia (Italy)	45.174	9.166	H32	LN552061	3	/
Hv22	*East/South-east-north*	Mantova (Italy)	45.168	10.807	H32	LN552066	3	/
Hv44	*East/South-east-north*	Aosta (Italy)	45.728	7.301	H31	LN552088	3	/
Hv5	*East/South-east-north*	Lentiai (Italy)	46.048	12.025	H32	LN552049	3	/
Hv34	*West*	Tombolo (Italy)	45.646	11.837	H1	LN552078	3	/

Hv27	*East/South-east-north*	Valle Strona (Italy)	45.608	8.811	H31	LN552071	3	/
Hv28	*West*	Asti (Italy)	44.894	8.235	H1	LN552072	3	/
Hv43	*West*	Alessa/ria (Italy)	44.912	8.593	H1	LN552087	3	/
Hv21	*East/South-east-north*	Montenovo (Italy)	44.073	12.301	H32	LN552065	3	/
Hv40	*East/South-east-north*	Monzuno (Italy)	44.278	11.274	H32	LN552084	3	/
Hv42	*East/South-east-north*	San Bartolomeo in Bosco (Italy)	44.735	11.637	H32	LN552086	3	/
Hv4	*West*	Pisa (Italy)	43.696	10.349	H1	LN552048	3	/
Hv19	*West*	Capraia (Italy)	43.029	9.827	H1	LN552063		/
Hv30	*West*	Campi Bisenzio (Italy)	43.829	11.138	H1	LN552074	3	/
Hv35	*West*	Calci (Italy)	43.714	10.5	H1	LN552079	3	/
Hv31	*West*	Lamone (Italy)	43.857	10.297	H1	LN552075	3	/
Hv45	*West*	Asinara (Italy)	41.081	8.293	H1	LN552089	3	/
Hv6	*West*	Sefro (Italy)	43.149	12.95	H1	LN552050	3	/
Hv10	*East/South-east-north*	Ancona (Italy)	43.605	13.543	H32	LN552054	3	/
Hv14	*East/South-east-north*	Ancona (Italy)	43.606	13.543	H32	LN552058	3	/
Hv8	*West*	Monterano (Italy)	42.138	12.099	H1	LN552052	3	/
Hv36	*West*	Roma (Italy)	41.913	12.482	H1	LN552080	3	/

Hv50	*East/South-east-north*	Pescara (Italy)	42.461	14.185	H32	LN552094	3	/
Hv2	*East/Central*	Pietraroja (Italy)	41.346	14.552	H21	LN552046	3	/
Hv7	*East/Central*	Avellino (Italy)	40.916	14.758	H21	LN552051	3	/
Hv11	*East/South-east-north*	Cava dei Tirreni (Italy)	40.695	14.717	H32	LN552055	3	/
Hv16	*East/Central*	Amalfi (Italy)	40.636	14.606	H21	LN552060	3	/
Hv18	*East/South-east-north*	Palinuro (Italy)	40.03	15.282	H32	LN552062	3	/
Hv51	*East/Central*	Soccavo (Italy)	40.852	14.206	H21	LN552095	3	/
Hv1	*East/South-east-north*	Lagonegro (Italy)	40.125	15.77	H29	LN552045	3	/
Hv9	*East/South-east-north*	Cetraro (Italy)	39.541	15.993	H32	LN552053	3	/
Hv12	*East/South-east-north*	Vallone Argentino (Italy)	39.428	16.126	H32	LN552056	3	/
Hv46	*East/South-east-north*	San Nicola Arcella (Italy)	39.843	15.797	H32	LN552090	3	/
Hv13	*East/South-east-north*	Bovino (Italy)	41.253	15.345	H32	LN552057	3	/
Hv24	*East/South-east-north*	Lecce (Italy)	40.356	18.147	H32	LN552068	3	/
Hv47	*East/Sicily*	Lago Spartà (Italy)	37.955	14.715	H14	LN552091	3	/

Hv20	*East/Sicily*	Iria (Italy)	38.038	14.637	H14	LN552064	3	/
Hv25	*West*	Blegiers (France)	44.18	6.416	H1	LN552069	3	/
Hv26	*West*	Montagnac la Crempx (France)	44.98	0.544	H1	LN552070	3	/
Hv29	*West*	Brusques (France)	43.772	2.944	H1	LN552073	3	/
Hv37	*West*	Castelnau Chalosse (France)	43.667	-0.846	H1	LN552081	3	/
Hv38	*West*	Millau (France)	44.105	3.046	H1	LN552082	3	/
Hv39	*West*	Larzac (France)	43.876	3.306	H1	LN552083	3	/
Hv48	*West*	Greolieres (France)	43.797	6.943	H1	LN552092	3	/
Hv49	*West*	Clumanc (France)	44.022	6.377	H1	LN552093	3	/
Hv23	*West*	Calanca (France)	41.651	8.894	H1	LN552067	3	/
Hv41	*West*	Biguglia (France)	42.622	9.444	H1	LN552085	3	/
Hv32	*East/South-east-north*	Obzova (France)	45.003	14.706	H27	LN552076	3	/
Hv3	*West*	Arties (Spain)	42.701	0.869	H1	LN552047	3	/
V24	*East/South-east-north*	Avio (Italy)	45.727	10.945	H32	FJ430636	4	/
V19	*East/South-east-north*	Doberdò del Lago (Italy)	45.844	13.543	H25	FJ430655	4	/
V1	*East/South-east-north*	Cascina Settimo (Italy)	45.255	9.215	H32	FJ430644	4	/
V3	*East/South-east-north*	Ardenno (Italy)	46.167	9.651	H32	FJ430622	4	/

V18	*East/South-east-north*	Capiago Intimiano (Italy)	45.772	9.128	H32	FJ430651	4	/
V20	*East/South-east-north*	Travacò Siccomarino (Italy)	45.151	9.158	H32	FJ430633	4	/
V25	*East/South-east-north*	Limbiate (Italy)	45.597	9.114	H32	FJ430637	4	/
V26	*East/South-east-north*	Limbiate (Italy)	45.597	9.114	H32	FJ430638	4	/
V13	*East/South-east-north*	Lentiai (Italy)	46.048	12.025	H32	FJ430641	4	/
V14	*East/South-east-north*	Lentiai (Italy)	46.048	12.025	H32	FJ430643	4	/
V15	*East/South-east-north*	Lentiai (Italy)	46.049	12.025	H32	FJ430645	4	/
V16	*East/South-east-north*	Lentiai (Italy)	46.049	12.025	H32	FJ430646	4	/
V2	*West*	Voltaggio (Italy)	44.628	8.843	H1	FJ430648	4	/
V21	*East/South-east-north*	Briga Novarese (Italy)	45.739	8.447	H31	FJ430634	4	/
V42	*West*	Villavernia (Italy)	44.823	8.855	H12	FJ430660	4	/
V23	*East/South-east-north*	Comacchio (Italy)	44.69	12.176	H32	FJ430635	4	/

V27	*East/South-east-north*	Travo (Italy)	44.861	9.547	H32	FJ430639	4	/
V4	*West*	Isola di Montecristo (Italy)	42.341	10.318	H1	FJ430631	4	/
V6	*West*	Isola di Montecristo (Italy)	42.341	10.318	H1	FJ430630	4	/
V7	*West*	Isola di Montecristo (Italy)	42.341	10.318	H1	FJ430627	4	/
V8	*West*	Isola di Montecristo (Italy)	42.341	10.318	H1	FJ430632	4	/
V33	*West*	Pisa (Italy)	43.696	10.349	H1	FJ430652	4	/
V12	*West*	Asinara (Italy)	41.081	8.293	H1	FJ430642	4	/
V22	*West*	Asinara (Italy)	41.081	8.293	H1	FJ430640	4	/
V41	*West*	Teulada (Spain)	38.969	8.763	H9	FJ430659	4	/
V38	*West*	Roccamassima (Italy)	41.68	12.921	H4	FJ430658	4	/
V17	*East/Central*	Salerno (Italy)	40.687	14.759	H21	FJ430647	4	/
V35	*East/Central*	Avella (Italy)	40.961	14.61	H21	FJ430649	4	/
V36	*East/Central*	Amalfi (Italy)	40.636	14.606	H21	FJ430650	4	/
V39	*East/South-east-north*	Lago Laceno (Italy)	40.804	15.107	H26	FJ430656	4	/
V40	*East/South-east-north*	Lago di Lesina (Italy)	41.896	15.379	H32	FJ430657	4	/
V37	*East/Sicily*	Lago Spartà (Italy)	37.955	14.715	H14	FJ430653	4	/
V9	*West*	Chizé (France)	46.124	-0.356	H1	FJ430628	4	/
V10	*West*	Chizé (France)	46.124	-0.357	H1	FJ430629	4	/
V11	*West*	Chizé (France)	46.124	-0.357	H1	FJ430621	4	/
V28	*West*	/	42.057	9.029	H1	FJ430654	4	/

V29	*West*	Molinos (Spain)	41.603	0.685	H1	FJ430623	4		/
V30	*West*	Molinos (Spain)			H1	FJ430624	4		/
V31	*West*	Molinos (Spain)			H1	FJ430625	4		/
V32	*West*	Pobellà (Spain)	42.374	1.11	H1	FJ430626	4		/
A1	*West*	Montanuy (Catalonia. Spain)	42.46555	0.6941	H1	OR229541	this study	AR	HV02 1
A4	*West*	Latasa (Navarre. Spain)	42.95330	-1.8222	H1	OR229542	this study	AR	HV01 1
A6	*West*	Irun (Gipuzkoa. Spain)	43.32691	-1.8125	H1	OR229543	this study	AR	HV03 1
CP2	*West*	Tuixent (Catalonia. Spain)	42.22845	1.5699	H1	OR229544	this study	UB	CRBA-43061
CP4	*West*	Arties (Catalonia. Spain)	42.70085	0.8674	H1	OR229545	this study	UB	CRBA-43072
CP5	*West*	La Torre de Cabdella (Catalonia. Spain)	42.42647	0.9840	H1	OR229546	this study	UB	CRBA-43392
CP6	*West*	Bagà (Catalonia. Spain)	42.25451	1.8549	H1	OR229547	this study	UB	CRBA-52316
AR1	*West*	Escarrilla (Aragon. Spain)	42.73848	-0.3147	H1	OR229548	this study	fieldwork	/
AR2	*West*	Panticosa (Aragon. Spain)	42.72936	-0.2703	H1	OR229549	this study	fieldwork	/
AR3	*West*	Benasque (Aragon. Spain)	42.62993	-0.1076	H1	OR229550	this study	fieldwork	/
AR4	*West*	Torla (Aragon. Spain)	42.66256	0.5842	H1	OR229551	this study	fieldwork	/
C3	*West*	Salardú (Catalonia. Spain)	42.70715	0.8959	H1	OR229552	this study	MCB	MZB 2010-1425
C4	*West*	Torre de Cabdella (Catalonia. Spain)	42.42261	0.9819	H10	OR229553	this study	MCB	MZB 2014-1957

C5	*West*	Bergà (Catalonia. Spain)	42.10554	1.8431	H1	OR229554	this study	MCB	MZB 2014-1964-B y -T
C6	*West*	Gisclareny (Catalonia. Spain)	42.26934	1.8052	H1	OR229555	this study	MCB	MZB 2017-0156
C7	*West*	Bagà (Catalonia. Spain)	42.25679	1.8479	H1	OR229556	this study	MCB	MZB 2019-0703
G1	*West*	Aiako Harriak (Irun.Gipuzkoa. Spain)	43.31868	-1.7678	H1	OR229557	this study	fieldwork	/
G2	*West*	Aiako Harriak (Irun.Gipuzkoa. Spain)	43.31868	-1.7625	H1	OR229558	this study	fieldwork	/
G3	*West*	Aiako Harriak (Irun.Gipuzkoa. Spain)	43.33130	-1.7558	H1	OR229559	this study	fieldwork	/
G4	*West*	Aiako Harriak (Irun.Gipuzkoa. Spain)	43.33123	-1.7560	H1	OR229560	this study	fieldwork	/
G5	*West*	Aiako Harriak (Irun.Gipuzkoa. Spain)	43.33120	-1.7563	H1	OR229561	this study	fieldwork	/
G6	*West*	Aiako Harriak (Irun.Gipuzkoa. Spain)	43.33120	-1.7563	H1	OR229562	this study	fieldwork	/
G7	*West*	Aiako Harriak (Irun.Gipuzkoa. Spain)	43.33123	-1.7560	H1	OR229563	this study	fieldwork	/

G8	*West*	Aiako Harriak (Irun.Gipuzkoa. Spain)	43.32722	-1.7563	H1	OR229564	this study	fieldwork	/
G9	*West*	Aiako Harriak (Irun.Gipuzkoa. Spain)	43.32971	-1.7634	H1	OR229565	this study	fieldwork	/
G10	*West*	Aiako Harriak (Irun.Gipuzkoa. Spain)	43.3302	-1.7646	H1	OR229566	this study	fieldwork	/
G11	*West*	Aiako Harriak (Irun.Gipuzkoa. Spain)	43.33130	-1.7558	H1	OR229567	this study	fieldwork	/
G12	*West*	Aiako Harriak (Irun.Gipuzkoa. Spain)	43.33066	-1.7647	H1	OR229568	this study	fieldwork	/
G13	*West*	Aiako Harriak (Irun.Gipuzkoa. Spain)	43.30206	-1.7549	H1	OR229569	this study	fieldwork	/
G14	*West*	Aiako Harriak (Irun.Gipuzkoa. Spain)	43.31868	-1.7625	H1	OR229570	this study	fieldwork	/
IP3	*West*	Orx (France)	43.59037	-1.3866	H1	OR229571	this study	AR	/
IP5	*West*	Messanges (France)	43.80951	-1.3970	H1	OR229572	this study	AR	/
IP6	*West*	Messanges (France)	43.80573	-1.3945	H6	OR229573	this study	AR	/
IP8	*West*	Bayonne (France)	43.47307	-1.4811	H1	OR229574	this study	AR	/
IP9	*West*	Bayonne (France)	43.46740	-1.4801	H1	OR229575	this study	AR	/
N1	*West*	Cía (Navarre. Spain)	42.93523	-1.7567	H1	OR229576	this study	AR	/
N2	*West*	Arakil (Navarre. Spain)	42.91728	-1.8429	H1	OR229577	this study	AR	/
N3	*West*	Urritza (Navarre. Spain)	42.96165	-1.8292	H1	OR229578	this study	AR	/
N4	*West*	Udabe (Navarre. Spain)	42.97085	-1.8248	H1	OR229579	this study	AR	/

N5	*West*	Etxarren (Navarre. Spain)	42.91629	-1.8485	H1	OR229580	this study	AR	/
N7	*West*	Ostiz (Navarre. Spain)	42.92040	-1.6253	H1	OR229581	this study	AR	/
P1	*East/Sicily*	Parco Nazionale dell'Aspromonte (Italy)	38.2902	16.037	H14	OR229582	this study	CEFE	BEV.14548
P7	*West*	Calvi (Corse. France)	42.56765	8.7572	H1	OR229583	this study	CEFE	BEV.5
P10	*West*	Pirio (Tuarelli. Corse. France)	42.37545	8.7513	H8	OR229584	this study	CEFE	BEV.22
P14	*West*	Fango (Corse. France)	42.40176	8.6976	H1	OR229585	this study	CEFE	BEV.59
P15	*West*	Sagone (Corse. France)	42.11016	8.6865	H1	OR229586	this study	CEFE	BEV.857
P16	*West*	Aleria (Corse. France)	42.15085	9.5066	H1	OR229587	this study	CEFE	BEV.8264
P17	*West*	Route D81 (Corse. France)	42.46638	8.7566	H1	OR229588	this study	CEFE	BEV.8266
P18	*West*	Galéria (Corse. France)	42.41035	8.6486	H1	OR229589	this study	CEFE	BEV.8353
P20	*West*	Biguglia (Corse. France)	42.60742	9.4847	H1	OR229590	this study	CEFE	BEV.8562
P22	*West*	Palombaggia (Corse. France)	41.56552	9.2773	H8	OR229591	this study	CEFE	BEV.10933
P23	*West*	Losari (Corse. France)	42.63338	9.0058	H12	OR229592	this study	CEFE	BEV.11044
P24	*West*	Urtaca (Corse. France)	42.62197	9.1202	H1	OR229593	this study	CEFE	BEV.11051
P25	*West*	Golf de Sperone (Bonifacio. Corse. France)	41.37320	9.2102	H1	OR229594	this study	CEFE	BEV.11060
P27	*West*	Galéria (Corse. France)	42.41671	8.6584	H1	OR229595	this study	CEFE	BEV.11295
P28	*West*	Frassigna (Corse. France)	42.4495	8.8289	H1	OR229596	this study	CEFE	BEV.10149
P29	*West*	Galéria (Corse. France)	42.3518	8.6461	H1	OR229597	this study	CEFE	BEV.10892
P30	*West*	Aleria (Corse. France)	42.105	9.5236	H1	OR229598	this study	CEFE	BEV. T4452

P31	*West*	Travo (Corse. France)	41.90607	9.3919	H1	OR229599	this study	CEFE	BEV. T4453
P32	*West*	St Julien de Rodelle (France)	44.49945	2.6630	H1	OR229600	this study	CEFE	BEV.8
P37	*West*	Le Caylar (France)	43.85700	3.3176	H1	OR229601	this study	CEFE	BEV.13
P49	*West*	Clamensane (France)	44.3241	6.0692	H1	OR229602	this study	CEFE	BEV.280
P53	*West*	Florac (France)	44.31460	3.6183	H1	OR229603	this study	CEFE	BEV.6201
P64	*West*	Gréolières (France)	43.78712	6.9738	H1	OR229604	this study	CEFE	BEV.8143
P65	*West*	Andon (France)	43.78266	6.8475	H1	OR229605	this study	CEFE	BEV.8144
P66	*West*	St-Sauveur sur Tinée (France)	44.08873	7.0422	H1	OR229606	this study	CEFE	BEV.8145
P67	*West*	Rosignano Marittimo (Italy)	43.38033	10.481	H1	OR229607	this study	CEFE	BEV.8263
P68	*West*	Larzac (France)	44.09341	3.1171	H13	OR229608	this study	CEFE	BEV.8563
P69	*West*	Rochefort-en-Valdaine (France)	44.5164	4.8611	H1	OR229609	this study	CEFE	BEV.8873
P70	*West*	Brusques (France)	43.76797	2.9533	H1	OR229610	this study	CEFE	BEV.8897
P72	*West*	Commune de Montagnac la Crempx (Dordogne. France)	44.98050	0.5463	H1	OR229611	this study	CEFE	BEV.9093
P74	*West*	Millau (France)	44.09535	3.0466	H7	OR229612	this study	CEFE	BEV.10391
P75	*West*	Gatuzières (France)	44.17984	3.5104	H1	OR229613	this study	CEFE	BEV.11014
P76	*West*	Gatuzières (France)	44.17984	3.5104	H1	OR229614	this study	CEFE	BEV.11015
P77	*West*	Route D107 entre Blegiers et Prads-Haute-Bleone (France)	44.20317	6.4230	H1	OR229615	this study	CEFE	BEV.11025
P78	*West*	Castellane (France)	43.78525	6.7460	H1	OR229616	this study	CEFE	BEV.11026

P79	*West*	Orlu (France)	42.70100	1.8903	H1	OR229617	this study	CEFE	BEV.11059
P82	*West*	Larzac (France)	43.98176	3.1432	H1	OR229618	this study	CEFE	BEV.11525
P85	*West*	Massegros (France)	44.30779	3.1724	H1	OR229619	this study	CEFE	BEV.9251
P87	*West*	Bessières (France)	43.78675	1.5770	H1	OR229620	this study	CEFE	BEV.14443

Publications referred as numbers:

1. Senczuk, G.; Gramolini, L.; Avella, I.; Mori, E.; Menchetti, M.; Aloise, G.; Castiglia, R. No association between candidate genes for color determination and color phenotype in *Hierophis viridiflavus*, and characterization of a contact zone. *J. Zoolog. Syst. Evol*. **2021**, *59(3),* 748–759.
2. Avella, I.; Castiglia, R.; Senczuk, G. Who are you? The genetic identity of some insular populations of *Hierophis viridiflavus* s.l. from the Tyrrhenian Sea. *Acta Herpetol*. **2017**, 12, 209–214.
3. Mezzasalma, M.; Dall'Asta, A.; Loy, A.; Cheylan, M.; Lymberakis, P.; Zuffi, M.A.L.; Tomovic, L.; Odierna, G.; Guarino, F.M. A sisters' story: Comparative phylogeography and taxonomy of *Hierophis viridiflavus* and *H. gemonensis* (Serpentes, Colubridae). *Zool. Scr*. **2015**, *44*, 495–508.
4. Rato, C.; Zuffi, M.A.L.; Corti, C.; Fornasiero, S.; Gentilli, A.; Razzetti, E.; Scali, S.; Carretero, M.A.; Harris, D.J. Phylogeography of the European Whip Snake, *Hierophis viridiflavus* (Colubridae), using mtDNA and nuclear DNA sequences. *Amphib-reptil*. **2009**, *30*, 283– 289.

Collections referred with acronyms: UB - Universitat de Barcelona (Barcelona, Spain); MCB - Museu de Ciènces Naturals de Barcelona (Barcelona, Spain); CEFE - Collection of the Biogeography and Ecology of the Vertebrates team in Montpellier (Montpellier, France); AR - Aranzadi Science Collection (Donostia, Spain).

Table S2 – Model ranking obtained from R package ENMeval in order to address Maxent model parameterization. FC= Feature class, RM= Regularization multiplier, AUC= Area under the curve, OR10= omission rate of testing localities at the 10% training threshold, AICc = Akaike's Information Criterion.

	fc	rm	auc.train	cbi.train	auc.diff.avg	auc.diff.sd	auc.val.avg	auc.val.sd	cbi.val.avg	cbi.val.sd	or.10p.avg	or.10p.sd	or.mtp.avg	or.mtp.sd	AICc	delta.AICc	w.AIC	ncoef
6	LQHPT	0.5	0.792	1.000	0.007	0.008	0.789	0.008	0.998	0.001	0.108	0.013	0.000	0.000	811.981	0	1	89
37	L	3.5	0.665	0.872	0.006	0.005	0.665	0.006	0.854	0.055	0.098	0.013	0.000	0.000	843.009	3.102	0	3
40	LQH	3.5	0.753	1.000	0.008	0.006	0.752	0.008	0.997	0.001	0.100	0.018	0.000	0.000	823.131	11.150	0	19
27	LQ	2.5	0.689	0.897	0.006	0.005	0.690	0.006	0.883	0.060	0.099	0.011	0.000	0.001	839.233	27.252	0	5
39	LQ	3.5	0.669	0.861	0.006	0.005	0.672	0.009	0.849	0.075	0.101	0.011	0.000	0.000	842.382	30.400	0	4
44	Q	4	0.645	0.680	0.006	0.005	0.645	0.006	0.663	0.097	0.101	0.010	0.000	0.000	846.467	34.486	0	3
36	LQHPT	3	0.767	1.000	0.008	0.007	0.764	0.009	0.993	0.006	0.100	0.014	0.000	0.000	819.579	75.981	0	32
28	LQH	2.5	0.756	1.000	0.008	0.006	0.755	0.008	0.997	0.002	0.101	0.015	0.000	0.000	822.002.	100.209	0	23
35	LQHP	3	0.756	1.000	0.008	0.006	0.754	0.008	0.997	0.001	0.100	0.016	0.000	0.000	822.107	101.261	0	15
34	LQH	3	0.755	1.000	0.008	0.006	0.753	0.008	0.997	0.002	0.099	0.014	0.000	0.000	822.420	104.393	0	17
41	LQHP	3.5	0.754	1.000	0.008	0.006	0.752	0.008	0.996	0.002	0.100	0.017	0.000	0.000	822.833	108.518	0	18
47	LQHP	4	0.752	1.000	0.008	0.006	0.751	0.008	0.996	0.002	0.101	0.018	0.000	0.000	823.349	113.680	0	15
46	LQH	4	0.752	1.000	0.008	0.006	0.751	0.008	0.996	0.003	0.101	0.019	0.000	0.000	823.672	116.906	0	17
3	LQ	0.5	0.718	0.988	0.006	0.005	0.717	0.006	0.975	0.020	0.101	0.017	0.000	0.000	831.916	199.348	0	7
9	LQ	1	0.711	0.898	0.006	0.005	0.711	0.005	0.865	0.097	0.099	0.013	0.000	0.000	834.618	226.375	0	7
15	LQ	1.5	0.705	0.811	0.006	0.005	0.705	0.005	0.812	0.124	0.098	0.014	0.000	0.000	836.08	241.013	0	5
21	LQ	2	0.698	0.822	0.006	0.005	0.698	0.006	0.822	0.087	0.100	0.012	0.000	0.001	837.623	256.418	0	5
12	LQHPT	1	0.784	1.000	0.007	0.008	0.781	0.008	0.994	0.002	0.104	0.018	0.000	0.000	814.597	261.611	0	69
33	LQ	3	0.680	0.868	0.006	0.005	0.681	0.008	0.860	0.070	0.099	0.012	0.000	0.000	840.794	288.128	0	4
1	L	0.5	0.667	0.906	0.005	0.005	0.667	0.006	0.880	0.068	0.100	0.011	0.000	0.000	842.380	303.993	0	4
7	L	1	0.667	0.884	0.005	0.005	0.667	0.006	0.872	0.064	0.099	0.010	0.000	0.000	842.464	304.827	0	4
13	L	1.5	0.667	0.872	0.005	0.005	0.667	0.006	0.863	0.064	0.099	0.010	0.000	0.000	842.534	305.582	0	3
19	L	2	0.667	0.868	0.005	0.005	0.666	0.006	0.863	0.055	0.099	0.011	0.000	0.000	842.646	306.651	0	3
25	L	2.5	0.666	0.872	0.006	0.005	0.666	0.006	0.856	0.056	0.099	0.013	0.000	0.000	842.769	307.786	0	3
31	L	3	0.666	0.871	0.006	0.005	0.665	0.006	0.854	0.054	0.099	0.013	0.000	0.000	842.881	308.996	0	3
43	L	4	0.664	0.871	0.006	0.005	0.664	0.006	0.850	0.056	0.098	0.014	0.000	0.000	843.144	311.628	0	3
45	LQ	4	0.664	0.871	0.006	0.005	0.666	0.008	0.852	0.059	0.099	0.012	0.000	0.000	843.144	311.628	0	3
2	Q	0.5	0.657	0.818	0.005	0.005	0.657	0.005	0.783	0.036	0.100	0.011	0.000	0.000	845.618.	336.371	0	4
8	Q	1	0.658	0.802	0.005	0.005	0.657	0.006	0.782	0.039	0.099	0.011	0.000	0.000	845.709	337.276	0	4
14	Q	1.5	0.657	0.792	0.005	0.005	0.656	0.006	0.768	0.038	0.099	0.011	0.000	0.000	845.790	338.094	0	3
20	Q	2	0.655	0.771	0.005	0.005	0.655	0.006	0.749	0.048	0.100	0.011	0.000	0.000	845.906	339.246	0	3
26	Q	2.5	0.653	0.751	0.005	0.005	0.653	0.006	0.731	0.060	0.100	0.011	0.000	0.000	846.031	340.500	0	3
32	Q	3	0.651	0.734	0.005	0.005	0.651	0.006	0.711	0.076	0.100	0.011	0.000	0.000	846.166	341.854	0	3
38	Q	3.5	0.648	0.708	0.005	0.005	0.648	0.006	0.685	0.088	0.101	0.010	0.000	0.000	846.312	343.308	0	3
11	LQHP	1	0.777	1.000	0.007	0.006	0.775	0.007	0.998	0.001	0.103	0.012	0.000	0.000	816.189	420.839	0	35
18	LQHPT	1.5	0.776	1.000	0.007	0.008	0.774	0.008	0.992	0.005	0.103	0.021	0.000	0.000	816.642	466.146	0	50

4	LQH	0.5	0.773	1.000	0.006	0.006	0.771	0.007	0.996	0.002	0.103	0.013	0.000	0.000	816.737	475.618	0	40
24	LQHPT	2	0.773	1.000	0.007	0.008	0.769	0.008	0.992	0.006	0.101	0.019	0.000	0.000	817.754	577.335	0	42
30	LQHPT	2.5	0.770	1.000	0.008	0.007	0.766	0.008	0.992	0.007	0.102	0.016	0.000	0.000	818.752	677.060	0	39
10	LQH	1	0.766	1.000	0.007	0.007	0.765	0.008	0.995	0.003	0.100	0.018	0.000	0.000	818.835	685.419	0	31
17	LQHP	1.5	0.767	1.000	0.007	0.006	0.763	0.008	0.997	0.002	0.099	0.012	0.000	0.000	818.889	690.789	0	27
16	LQH	1.5	0.761	1.000	0.007	0.006	0.759	0.007	0.996	0.002	0.100	0.016	0.000	0.000	820.240	825.881	0	24
42	LQHPT	3.5	0.765	0.999	0.008	0.007	0.762	0.008	0.990	0.007	0.100	0.015	0.000	0.000	820.340	835.909	0	29
5	LQHP	0.5	0.789	1.000	0.007	0.005	0.787	0.007	0.998	0.001	0.103	0.009	0.000	0.000	812.823	842.299	0	51
23	LQHP	2	0.758	1.000	0.007	0.006	0.757	0.007	0.997	0.001	0.101	0.015	0.000	0.000	820.924	894.335	0	22
48	LQHPT	4	0.763	0.999	0.008	0.007	0.761	0.008	0.988	0.006	0.101	0.016	0.000	0.000	820.946	896.481	0	23
22	LQH	2	0.757	1.000	0.008	0.006	0.756	0.007	0.996	0.001	0.101	0.016	0.000	0.000	821.261	928.036	0	21
29	LQHP	2.5	0.757	1.000	0.008	0.006	0.756	0.008	0.997	0.001	0.103	0.015	0.000	0.000	821.774	979.303	0	26

Supplementary figures

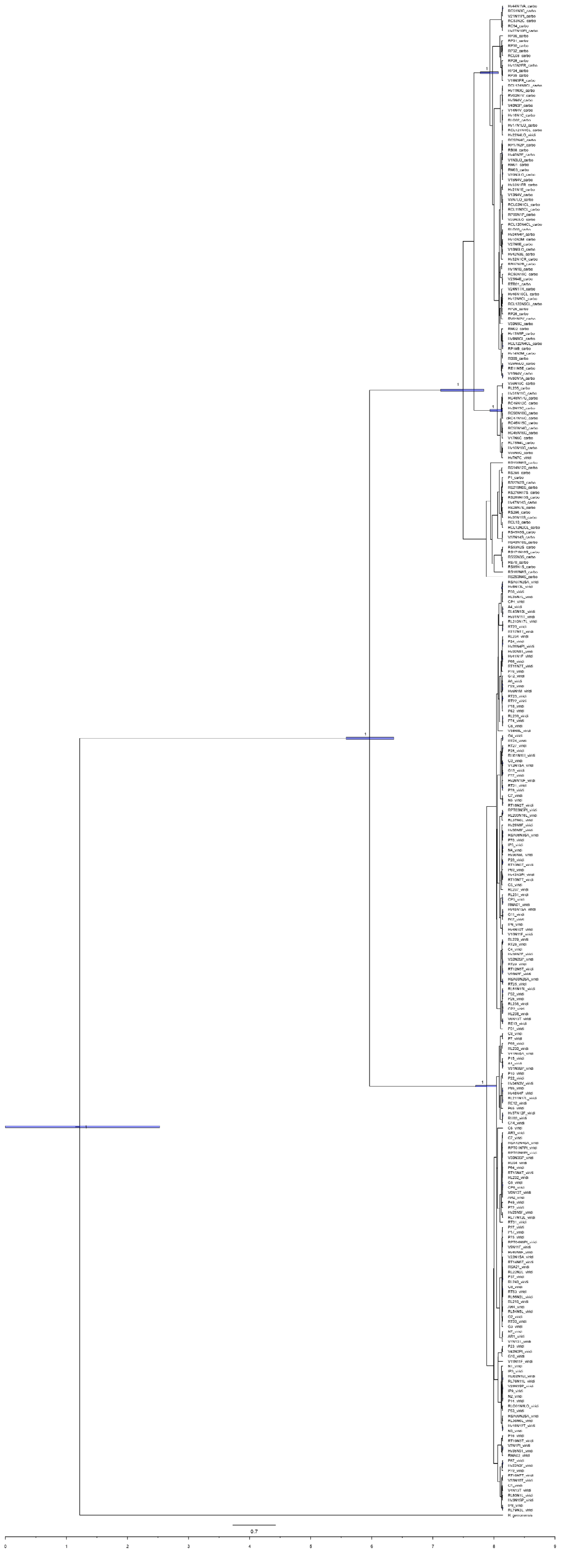

Figure S1. Calibrated BEAST phylogenetic tree with the codes of all sequences that were considered in the analysis, the values of node support and the node bars that indicate the divergence date 95% highest posterior density.

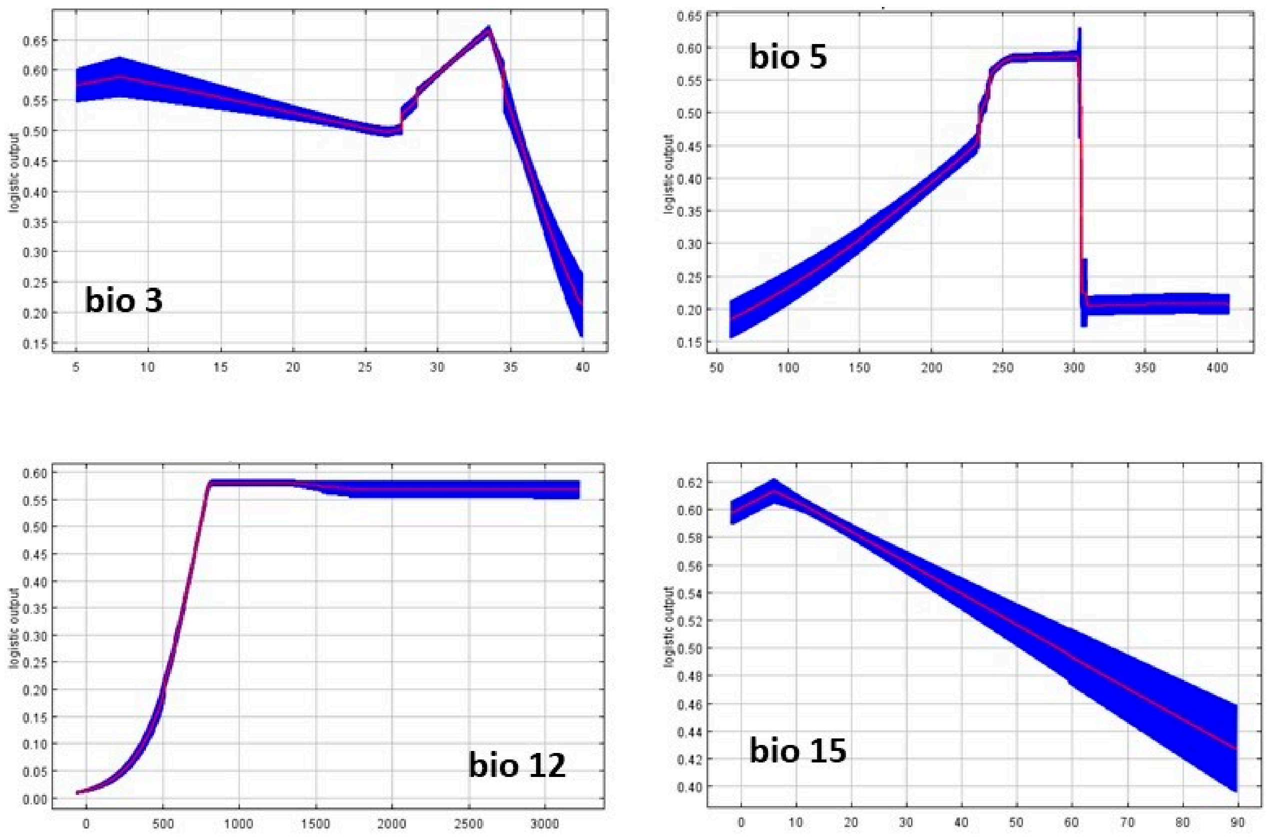

Figure S2. Average response curve profiles for the used variables. See table 2 for variables names.

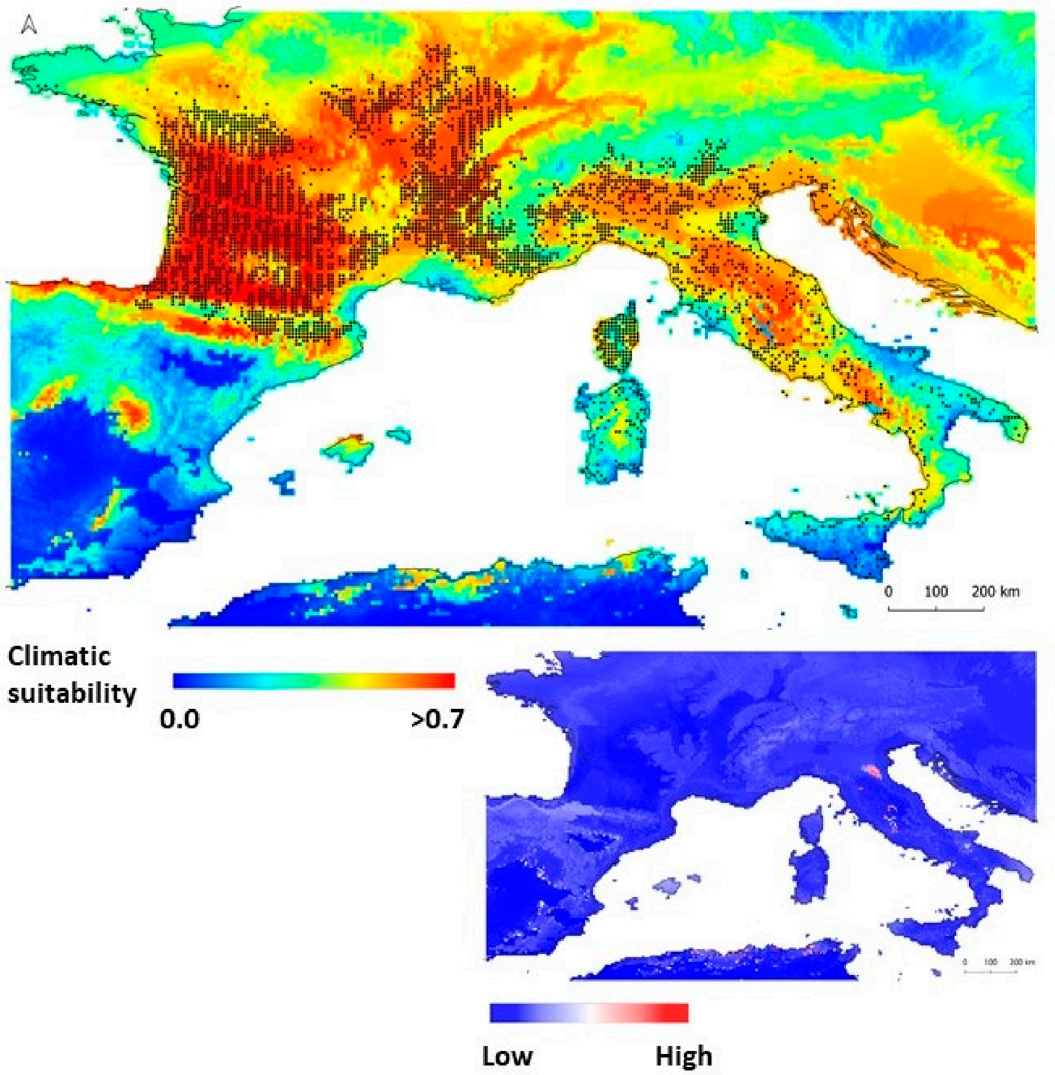

Figure S3. Average suitability (top) and SD model (bottom) for current conditions for *Hierophis viridiflavus*. Dots in the suitability map correspond to all samples used for modeling.

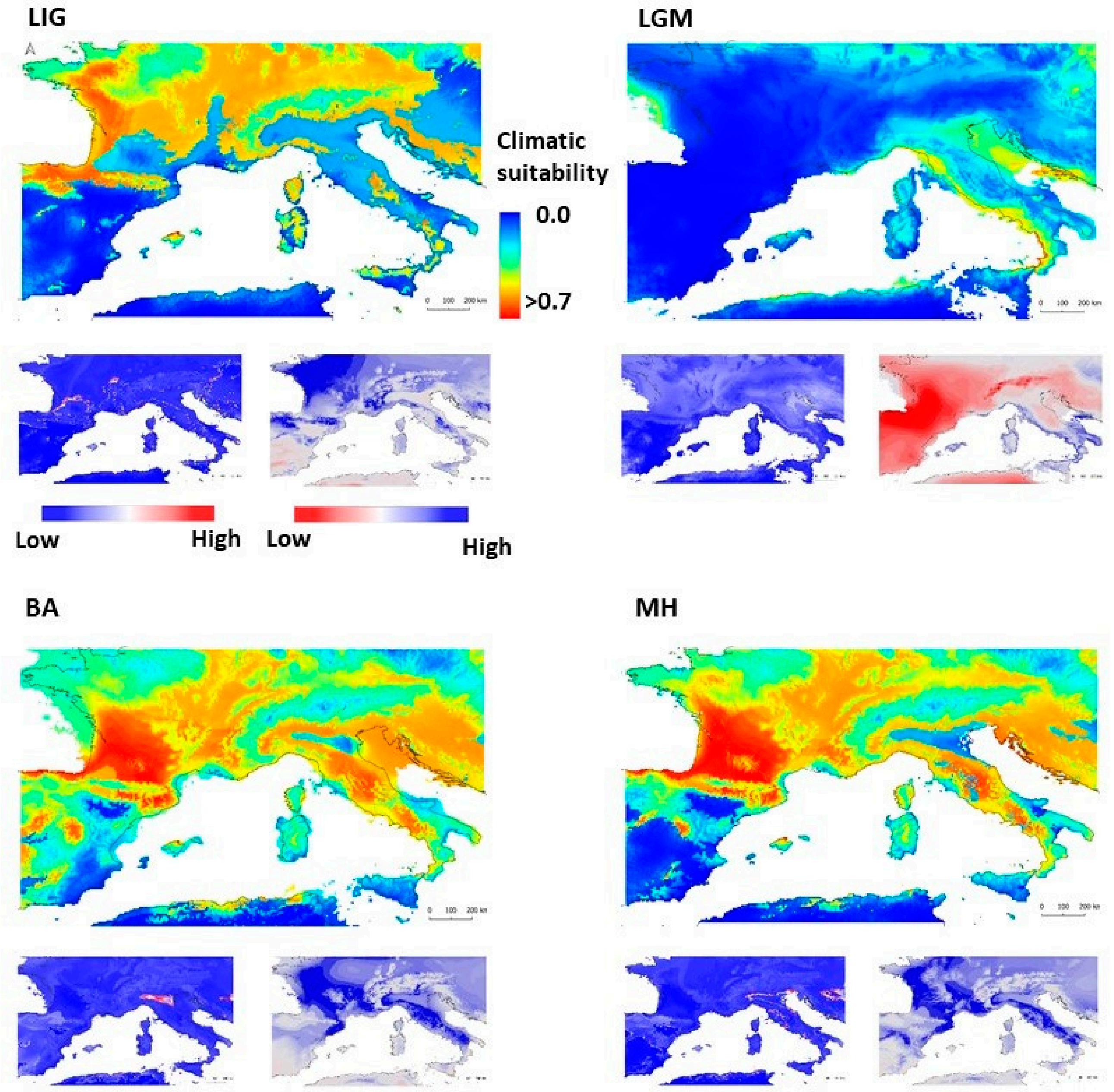

Figure S4. Average suitability projections (top), and SD (bottom left) and MESS (bottom right) plots, for four different past periods (LIG – Last Interglacial; LGM - Last Glacial Maximum; BA - Bølling-Allerød; MH - mid Holocene) for *Hierophis viridiflavus*.
